# Reinforcement Learning-Driven Autonomous Path Planning for Unmanned Surface Vehicles: Current Status, Challenges, and Future Prospects

**DOI:** 10.3390/s26092852

**Published:** 2026-05-02

**Authors:** Zexu Dong, Jiashu Zheng, Chenxuan Guo, Fangming Zhao, Yijie Chu, Xiaojun Chen

**Affiliations:** FedUni Information Engineering Institute, Hebei University of Science and Technology, Shijiazhuang 050018, China; 2401602118@stu.hebust.edu.cn (Z.D.); 2401601112@stu.hebust.edu.cn (J.Z.); 065003@hebust.edu.cn (C.G.); 2501604223@stu.hebust.edu.cn (F.Z.)

**Keywords:** unmanned surface vehicles, local path planning, reinforcement learning, collision avoidance, deep reinforcement learning, sim-to-real transfer

## Abstract

The continuous advancement of autonomy and intelligence in marine shipping has made the safe and efficient navigation of unmanned surface vehicles in complex waters a major research focus. As a key link of the autonomous decision-making system for unmanned surface vehicles (USVs), local path planning needs to achieve real-time collision avoidance and motion optimization under dynamic obstacles, multiple rule constraints, and strong environmental uncertainty. In recent years, reinforcement learning has gradually become an important technical route for local path planning of USVs by virtue of its autonomous decision-making ability in high-dimensional continuous state space and adaptability to complex nonlinear problems. Combined with the evolution of the algorithm paradigm and its functional positioning in different water scenarios, this paper systematically reviews the relevant literature by examining the evolution of algorithmic paradigms; focuses on summarizing deep Q-network (DQN), Proximal Policy Optimization (PPO), Soft Actor-Critic (SAC), and Twin Delayed Deep Deterministic Policy Gradient (TD3), along with the collaborative architectures integrated with traditional planning methods such as A* and Rapidly-exploring Random Tree (RRT); and summarizes the performance characteristics, advantages, and limitations of various methods in typical scenarios. The review shows that the main bottlenecks of current research include insufficient reward mechanism design, low sample utilization efficiency, difficulty in transferring from simulation to real ships, and insufficient safety and trustworthiness verification. This paper looks forward to the future development trends from the two directions of data fusion and security enhancement in order to provide reference for related research.

## 1. Introduction

The rapid development of autonomous unmanned system technology has made USVs an important platform for performing water-based tasks. As the core of the autonomous navigation system, its path planning technology directly determines the success rate of the task and navigation safety [1,2]. To date, its application scenarios have mainly focused on the open sea [3,4,5,6], whereas narrow waterways, busy ports, inland rivers and lakes, and complex reef waters have not yet been fully explored. Complex water environments are difficult to perceive and impose strong navigation constraints, which place higher requirements on path planning. At present, there are still many technical challenges, and no large-scale application has been achieved. However, it can be predicted that overcoming the problems of autonomous navigation in these complex scenes is an important direction for the development of USV technology, and it is also an inevitable trend to realize its full water application.

Traditional path planning methods, such as the A* algorithm, the artificial potential field method (APF), and RRT, show good computational efficiency under static environments or simple rule constraints [7,8]. However, in the face of complex dynamic flow fields (such as eddies and linear flows), frequent dynamic and static obstacles, and strict constraints of the International Regulations for Collision Avoidance at Sea (COLREGs) in real waters, traditional algorithms often face challenges such as the curse of dimensionality, a tendency to fall into local optima, and a lack of environmental adaptability [5,9,10]. In view of this, reinforcement learning (RL) provides a new paradigm for path planning of USVs in complex waters with its powerful nonlinear mapping ability and the characteristics of “obtaining the optimal policy through trial and error in large-scale state space” [6,11].

The application of reinforcement learning in the field of USV has so far shown a high degree of interdisciplinary integration. By introducing DQN, PPO, and SAC and their various improved variants, researchers have not only improved the success rate of collision avoidance of the algorithm in complex waters [12,13,14]. Moreover, the frontier issues such as energy efficiency optimization, multi-task collaboration, and Sim-to-Real cross-domain migration are further discussed [4,15,16]. In addition, for the special needs of specific waters, such as attitude control in narrow passages, multi-agent cooperation in port environments, and path correction in eddy environments, a series of reinforcement learning frameworks combining attention mechanisms, memory space, and dynamic model enhancement have been proposed [5,17,18,19].

Several recent review studies have provided important references for this field, but they differ in scope and emphasis. Li et al. systematically reviewed deep reinforcement learning methods in USV collision avoidance, focusing on value-based, policy-based, and multi-agent DRL methods, and summarized key issues such as reward design, state space representation, safety rules, and disturbance [20]. Venu and Gurusamy provided a more macro overview of path planning for autonomous navigation, covering classical, metaheuristic, and AI-based approaches but do not specifically focus on RL-driven path planning for USVs [21]. Yu et al. reviewed the application of reinforcement learning in Autonomous Underwater Vehicle (AUV) motion systems and organized it by motion control, motion planning, and multi-AUV motion, which is different from the perspective of surface vehicle path planning focused on this paper [22]. In addition, Heng et al. reviewed the application of ant colony optimization in USV path planning, emphasizing a single biological heuristic optimization method, rather than the broader RL-based algorithm evolution that is the focus of this paper [23]. From the existing research, the systematic review of RL-driven local path planning for USVs, the evolution of representative algorithms, and the synergistic relationship with traditional planning methods are still relatively insufficient.

**Figure 1:**
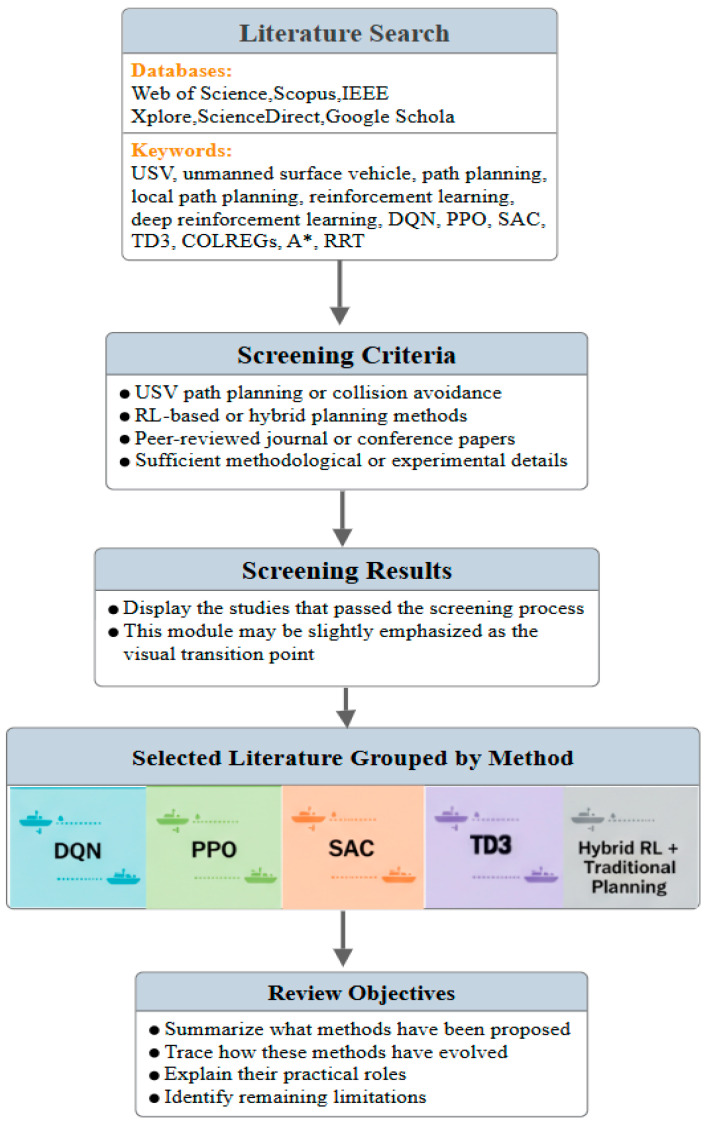
Framework for literature search, screening, and classification.

Based on the above research background, this paper focuses on RL-driven local path planning for USVs and adopts a classification strategy that combines algorithm evolution with functional positioning to systematically organize the application of different methods across typical water scenarios. As shown in Figure 1, the literature review process in this study consists of three stages: literature retrieval, screening, and classification. The retrieval sources include Web of Science, Scopus, IEEE Xplore, ScienceDirect, and Google Scholar, while the screening criteria mainly consider relevance, methodological type, publication venue, and the completeness of methodological and experimental information. On this basis, this paper further summarizes the application modes, performance characteristics, advantages, and limitations of representative RL algorithms in USV path planning and identifies common issues in current research, including training stability under sparse-reward conditions, effective integration of multiple navigation rules, and insufficient robustness in complex real-world sea conditions. Finally, future development directions are discussed from the perspectives of data fusion and safety enhancement.

## 2. Current Research

In recent years, the application of reinforcement learning in local path planning of USVs has continued to expand, and the research focus has been gradually extended from the early single obstacle avoidance decisions to trajectory optimization under complex constraints, navigation rule fusion, energy efficiency coordination, and transfer from simulation to real ships [2,8]. Compared with traditional planning methods, reinforcement learning can not only realize online responses in dynamic environments but also deal with environmental perception, action decisions, and task constraints simultaneously in a unified framework, thus providing a more adaptive solution for autonomous navigation in complex waters.

From the methodological level, existing studies can be summarized according to the algorithmic paradigm and its functional positioning in the USV path planning task. According to the logical expansion from the value-function-based method to policy optimization, and then to the hybrid planning architecture, this paper reflects the method evolution and engineering application context of reinforcement learning in USV path planning. DQN mainly corresponds to discrete decision problems driven by value functions, which are suitable for scenarios with relatively clear action spaces and regular control logic. As a stable policy optimization method, PPO has outstanding performance in training convergence and engineering realizability. SAC enhances the exploration ability and robustness through the maximum entropy mechanism, which is suitable for decision-making optimization in complex dynamic environments. TD3 is an important improvement method in continuous control scenarios, which is mainly used to alleviate value overestimation and improve control accuracy. In addition, the hybrid architecture combining reinforcement learning with traditional planning methods such as A* and RRT* has gradually become an important idea to balance global feasibility and local responsiveness. These methods do not replace each other completely but correspond to different control requirements, environmental complexity, and system constraints and eventually forming complementary technical systems.

**Figure 2:**
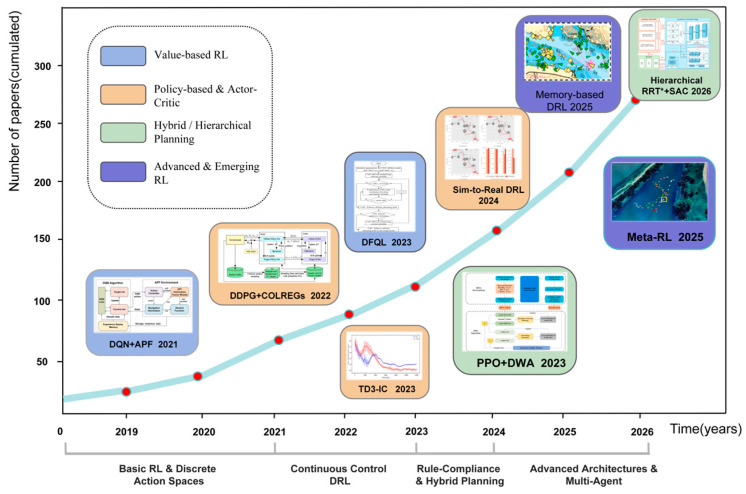
The evolutionary timeline and representative milestones of deep reinforcement learning (DRL) for USV path planning.

Figure 2 presents the development timeline and cumulative publication trends of representative reinforcement learning methods in USV path planning. It highlights not only the rapid increase in research interest but also the algorithmic paradigm shifts across four main phases: early discrete-action exploration, continuous control DRL, rule-compliant hybrid planning, and advanced multi-agent architectures [24]. Following this developmental logic, the subsequent sections will detail the application of core algorithms—including DQN, PPO, SAC, TD3, and hybrid structures—analyzing their underlying mechanisms, performance trade-offs, and typical use cases.

### 2.1. Research Status of the DQN Algorithm in USV Path Planning

**Figure 3:**
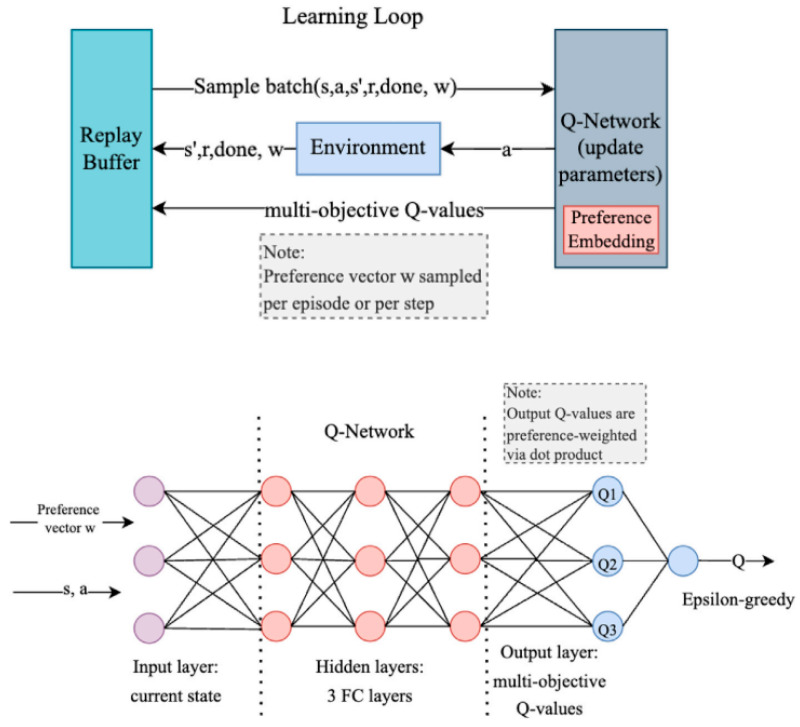
Illustration of the architecture of MODQN [12].

The DQN algorithm uses a deep neural network to approximate the Q(s,a) value function, employs experience replay to break the temporal correlation among samples, and relies on a target network to improve training stability [12,18]. In USV path planning, it typically maps the environmental state into a discrete action set, such as turning left, turning right, or moving straight. Figure 3 illustrates the architecture of the multi-objective deep Q-network (MODQN), in which the current environmental state is processed through multiple fully connected layers to extract features and generate multiple Q-values corresponding to objectives such as energy consumption, safety, and path efficiency. Preference-vector weighting is then used to balance multiple objectives, while the experience replay mechanism further improves training performance and stability [12].

#### 2.1.1. Advantages of the DQN Algorithm in USV Path Planning

- In the scenario where the environmental structure is relatively regular and the action space can be discretized, DQN can learn the value relationship between the state and the action more stably, so that the USVs can gradually form a repeatable obstacle avoidance and navigation strategy, which is suitable for path planning tasks in known waters such as ports and inland rivers [18,25].
- The experience replay mechanism enables the algorithm to reuse historical navigation samples multiple times, resulting in high sample utilization efficiency and favorable convergence characteristics when relatively complete prior environmental information is available [25]. This mechanism also helps break the temporal correlation between consecutive samples, thereby improving training stability and reducing the risk of catastrophic forgetting [12].
- In static or weak dynamic obstacle scenarios, DQN-based path planning methods show good decision stability and are often used for offline path planning verification and preliminary studies of rule-driven obstacle avoidance methods [26].

#### 2.1.2. Disadvantages of the DQN Algorithm in USV Path Planning

- Due to the discrete modeling of the action space, the USV needs to frequently switch course or rudder angle commands when executing the planned path, which leads to the generated trajectory exhibiting zigzag patterns and insufficient smoothness, thereby compromising maneuverability and energy efficiency in actual navigation [25]. This issue is particularly evident in narrow channels where frequent rudder angle adjustments are required [3,17].
- In dynamic collision avoidance tasks with high-speed or highly maneuvering targets, the inherent value function overestimation problem of DQN may affect the assessment of collision risk of USVs, which makes the planning strategy deviate from the safety margin, thus reducing the reliability of decision-making [9].
- When continuous speed control, flow field information, or multi-target joint perception features are introduced, the dimension of state space is significantly increased, and the training efficiency and convergence performance of DQN are easily affected, which limits its direct application in complex real water environments [5,12].

In recent years, aiming to address the issue that the traditional DQN path is not smooth enough, some studies have introduced Radial Basis Function (RBF) networks to approximate the value function and combined them with spline curves to quadratically smooth the trajectory in the post-processing stage so that the planned path is more in line with the physical manipulation characteristics of USVs [25]. In terms of multi-task collaboration, the multi-objective deep reinforcement learning framework is used in restricted waters such as busy ports to achieve multi-objective trade-offs by decoupling safety, energy efficiency, and path cost [12]. In addition, for the water environment containing eddy currents and DC, researchers have constructed an intelligent planning system with the characteristics of “downstream energy saving” by integrating the flow field characteristics into the state space [5]. Overall, DQN is more suitable for scenarios with discretization of action space, regular environmental structure, and relatively low task complexity, especially for basic obstacle avoidance and rule-driven navigation tasks in ports or inland rivers.

### 2.2. Research Status of the PPO Algorithm in USV Path Planning

**Figure 4:**
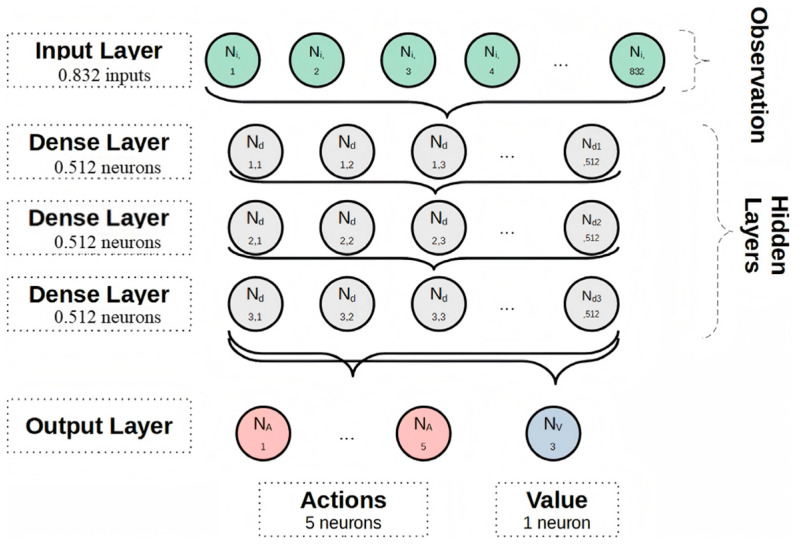
Illustration of the neural network architecture for the PPO algorithm [14].

The PPO algorithm is a policy gradient algorithm based on Actor-Critic architecture. The proximal clipping mechanism is introduced to limit the ratio of new and old policies to the interval [1 − ϵ, 1 + ϵ], which ensures that policy updates proceed smoothly in a reasonable trust domain and avoids gradient explosion or policy collapse [14,27,28]. As shown in Figure 4, the PPO neural network architecture typically consists of an actor subnet and a critic subnet. The actor subnet generates action decisions based on the environmental state to adjust heading and speed, whereas the critic subnet estimates the value function of the current state and provides guidance for policy optimization. This dual-network structure contributes to improved training stability and continuous control capability [14].

#### 2.2.1. Advantages of the PPO Algorithm in USV Path Planning

- It can directly output continuous action commands, such as rudder angle and propulsion speed, which can generate smooth tracks in USV path planning tasks and better meet the requirements of ship maneuverability and kinematic constraints [26]. This capability is particularly valuable in narrow channels where smooth trajectory execution is critical for safe passage [3,17].
- It has good robustness in task scenarios with multiple constraints, especially when the international rules for COLREGs and attitude constraints are integrated; the algorithm shows relatively stable convergence characteristics [14].

#### 2.2.2. Disadvantages of the PPO Algorithm in USV Path Planning

- Typical online policy optimization algorithms have low sample utilization, and historical samples are difficult to reuse after policy updating. In complex environments, such algorithms often require large computational overhead [6].
- The performance of the algorithm is sensitive to the design of the reward function. When the reward logic is not constructed properly, the local optimal solution of “effective collision avoidance behavior but redundant path” is easy to appear, which affects the overall navigation efficiency [29]. In low-visibility inland river environments, poorly designed reward structures may lead to overly conservative behaviors that compromise navigation efficiency.

At the local collision avoidance decision level, PPO has become the mainstream scheme for integrating maritime rules. The researchers embedded the international rules for COLREGs as a logical penalty item in PPO training and realized autonomous navigation decision-making to meet the legal requirements, such as “right-rudder avoidance” in crowded waters [14]. Aiming at the problem of narrow channel perception, the dual attention mechanism is embedded in the PPO architecture, which adaptively focuses on targets with high collision risk, significantly improving the efficiency of USVs in narrow corridors [3,17,25]. In the inland river environment with very low visibility, the perception-decision integration model based on PPO enhances the system’s ability to resist environmental noise by fusing the Collision Risk Index (CRI) [29]. Overall, PPO is more suitable for scenarios requiring continuous control, dynamic collision avoidance, and compliance with COLREGs constraints, particularly for narrow-channel passage and rule-constrained navigation tasks.

### 2.3. Research Status of the SAC Algorithm in USV Path Planning

**Figure 5:**
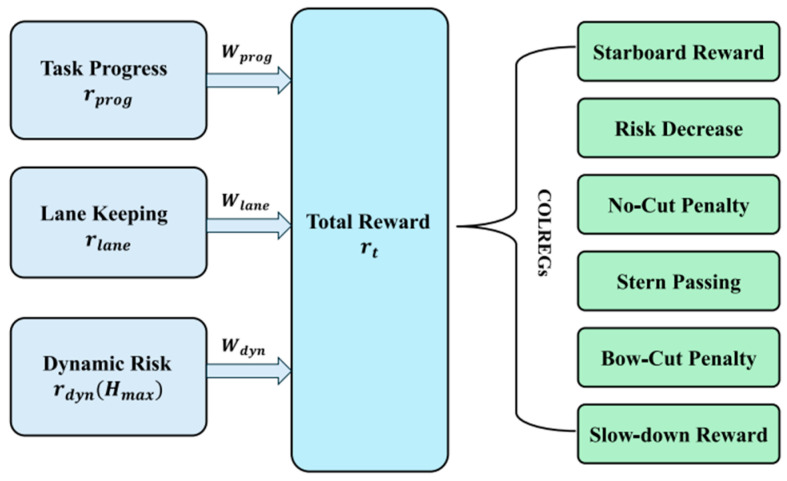
Schematic representation of the reward function for SAC local path planning [30].

As shown in Figure 5, the SAC algorithm is based on the maximum entropy reinforcement learning framework. Its core idea is to introduce a policy entropy regularization term on top of maximizing cumulative reward, enabling the agent to maintain high exploration randomness during learning and thereby improve adaptability in unknown waters [9,30]. In USV local path planning, however, the effectiveness of SAC depends not only on the entropy mechanism itself but also heavily on reward design. The reward function determines whether the policy can simultaneously balance task progress, collision risk, path smoothness, and COLREGs compliance. Therefore, Figure 5 does not merely list isolated reward items; instead, it illustrates a key training mechanism in SAC-based USV local path planning, including task progress reward, dynamic risk assessment, and incentives and penalties related to COLREGs rules such as fairway holding, deceleration, and trailing passage [30].

#### 2.3.1. Advantages of the SAC Algorithm in USV Path Planning

- Through the introduction of the maximum entropy objective function, the strategy can effectively alleviate the problem of premature convergence to the local optimum and show good generalization ability in the waters with fuzzy geographical features or unstructured features (such as islands, reefs areas, and complex harbor environments) [31].
- Due to the randomness of the strategy itself, the algorithm shows certain robustness and strong adaptability to external disturbances in the face of uncertain environmental noise such as wind and wave disturbances and water flow changes [9].

#### 2.3.2. Disadvantages of the SAC Algorithm in USV Path Planning

- The algorithm structure is complex, usually, including multiple network branches and an entropy coefficient automatic adjustment mechanism, which puts forward high requirements for the computing resources and real-time performance of the embedded control platform. It is difficult to deploy in the USV systems with limited computing power [9,30]. This computational burden can lead to delayed decision-making in time-critical scenarios, such as high-speed pursuit evasion tasks [27,32].
- In a single water scene with clear channel structure and strong environmental certainty, the continuous exploration characteristics of the strategy may introduce unnecessary action disturbances, resulting in slight yaw or redundant adjustments of the track [30]. Such behavior can reduce energy efficiency and increase wear on actuators during routine navigation in well-structured inland waterways or regular shipping lanes.

For robust navigation in real complex sea conditions, researchers have integrated Hamiltonian Monte Carlo (HMC) sampling optimization into the SAC framework, which solves the trajectory swing problem under strong disturbances and improves the stability of the execution path [9]. In the hierarchical planning architecture, SAC is often used as a local action generator and cooperates with the global RRT* algorithm to deal with dynamic collision avoidance while ensuring global optimality [30,31]. In addition, SAC has been used to improve the accuracy of active obstacle avoidance and covert path generation of USVs in highly dynamic tasks such as underwater target observation or pursuit evasion [27,32]. Overall, SAC is more suitable for navigation tasks with strong uncertainty, complex disturbances, and continuous control demands, particularly in highly dynamic and unstructured marine environments.

### 2.4. Research Status of the TD3 Algorithm in USV Path Planning

**Figure 6:**
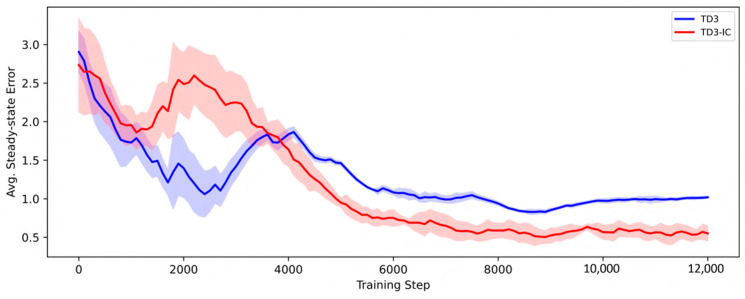
Curve of the steady-state error as a function of the number of steps in the training phase of TD3 [34].

The TD3 algorithm makes three major improvements to solve the overestimation bias in the Deep Deterministic Policy Gradient (DDPG) architecture: using a twin critic network to minimize the overestimation bias, implementing delayed policy updates, and introducing a target policy smoothing technique. These improvements make it perform better when dealing with the USV systems with strong inertia and strong nonlinear characteristics [33,34]. As shown in Figure 6, the average steady-state error gradually decreases with the increase in training steps and eventually tends to stabilize, indicating that TD3 can achieve accurate and stable policy learning in the speed and heading control of USVs.

#### 2.4.1. Advantages of the TD3 Algorithm in USV Path Planning

- The two-critic structure effectively alleviates the systematic bias in value function evaluation and has high decision reliability in the fine motion control task of underactuated USV systems [33,34]. Its delayed policy update and target smoothing mechanisms further help suppress control oscillations and improve execution stability under disturbance, which is particularly critical in scenarios requiring sustained precise heading and speed regulation, such as narrow channel passage [3,17].
- In long-endurance navigation and high-dynamic obstacle environments, TD3 shows stronger stability and robustness than the traditional DDPG algorithm, which is suitable for path planning and collision avoidance tasks with high requirements for control accuracy and safety margin [33].

#### 2.4.2. Disadvantages of the TD3 Algorithm in USV Path Planning

- The algorithm is sensitive to the accuracy and delay of the state returned by the sensor, and the convergence efficiency decreases when facing extreme fuzzy environmental perception [33,34]. In environments with inherently incomplete or noisy observations, such as low-visibility inland rivers or complex reef waters, this sensitivity may significantly degrade path planning reliability and control accuracy [9,29].

In recent years, aiming at the trajectory following problem of underactuated USVs under wind and wave disturbances, researchers combined integral compensation with the TD3 algorithm, which effectively canceled the real-time prediction residual caused by sideslip and inertia, and realized the path deviation control of centimeter level [33,34]. In the Sim-to-Real cross-domain research, the Meta-RL framework is introduced into TD3 so that the USVs can quickly transfer the obstacle avoidance strategy learned in the simulation environment to the real ship test and greatly shorten the strategy adaptation period [16,35]. For the multi-ship cooperative scenario, TD3 is combined with the belief assignment logic to solve the problems of real-time collision warning and cooperative obstacle avoidance in formation navigation [6,19]. Overall, TD3 is more suitable for complex tasks that require high-precision continuous control, cross-domain transfer capability, and cooperative obstacle avoidance in multi-agent settings.

### 2.5. Research Status of Hybrid Reinforcement Learning and Collaborative Planning Architecture in USV Path Planning

**Figure 7:**
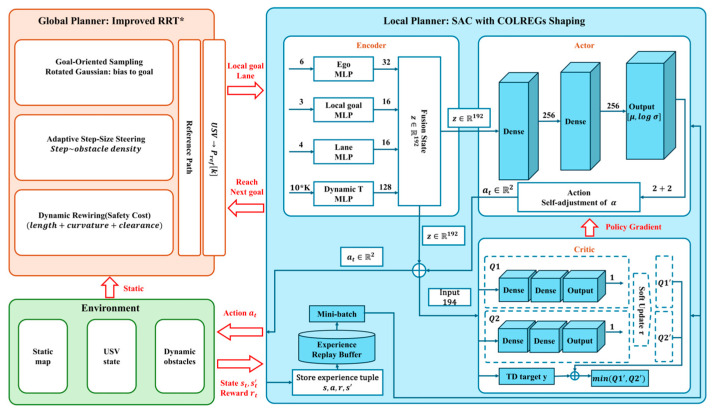
Illustration of the hybrid reinforcement learning and collaborative planning architecture [30].

The core idea of the hybrid reinforcement learning and collaborative planning architecture is to combine the advantages of reinforcement learning in dynamic perception and online decision-making with the ability of traditional path planning algorithms (such as A*, RRT*, artificial potential field method, etc.) in global search and feasibility guarantee to build a composite decision-making system of hierarchical planning or collaborative optimization [1,36]. In this kind of architecture, traditional algorithms are usually responsible for generating a global reference path that satisfies the static environmental constraints, while reinforcement learning algorithms dynamically modify the path within the local perception scope to cope with real-time changes in obstacles and environmental disturbances. As shown in Figure 7, the hierarchical hybrid path planning framework integrates the RRT* global planner with the SAC local controller, where the global planner provides a feasible reference path and the local controller performs adaptive local adjustment for dynamic obstacle avoidance and path tracking [30].

#### 2.5.1. Advantages of Hybrid Reinforcement Learning and Collaborative Planning Architecture in USV Path Planning

- By providing global path constraints by traditional algorithms, the exploration difficulty of reinforcement learning under sparse reward conditions can be effectively reduced, and the learning process is more stable [7,8]. At the same time, reinforcement learning is responsible for dealing with local burst risks and dynamic collision avoidance so as to achieve both global optimality and local responsiveness.
- In the case of abnormal perception or failure of learning strategy, it can switch to the backup traditional planning algorithm so as to maintain the basic obstacle avoidance and navigation logic, improving the overall safety and engineering reliability of the system [31].

#### 2.5.2. Disadvantages of Hybrid Reinforcement Learning and Collaborative Planning Architecture in USV Path Planning

- Different algorithm modules involve the transformation of state expression, control interface, and decision timing; and their execution weight allocation and co-scheduling logic are complex. If not properly designed, it is easy to cause conflicts between local decisions and global planning, affecting system consistency and real-time performance [30].

The current development trend focuses on the “benchmark path + dynamic correction” mode. The improved genetic algorithm, or A* algorithm, is used to plan the long-term path that meets the requirements of static waters, and then the reinforcement learning algorithm is used to fine-tune the path according to COLREGs rules in the local perception area [35,36]. In addition, deep reinforcement learning is also combined with multi-layer boundary semantic segmentation technology to solve the boundary intrusion risk of USVs in the fuzzy waters of the bank line by optimizing the accuracy of the environment representation [37]. Overall, this hybrid paradigm demonstrates strong engineering potential in highly complex scenarios such as confluence waters, narrow channels, and environments with unclear boundaries [38].

The advantages and disadvantages of the above commonly used reinforcement learning algorithms for USV local path planning are summarized in Table 1, while Table 2 provides a five-dimensional performance comparison of representative reinforcement learning and hybrid planning methods.

In general, the research on reinforcement learning in local path planning of unmanned ships has formed a relatively clear methodological vein. DQN provides basic ideas for discrete action decision-making; PPO performs outstandingly in training stability and engineering realizability; SAC enhances exploration ability and robustness through a maximum entropy mechanism; and TD3 further improves action accuracy and learning stability in continuous control tasks. Additionally, hybrid architecture reflects the research trend of collaborative integration of reinforcement learning and traditional planning methods. Although different methods have different focuses, they all focus on the path generation, obstacle avoidance control, and autonomous decision-making of USVs in complex environments and together constitute an important methodological basis for the current path planning research of USVs.

## 3. Problems and Challenges

Although reinforcement learning has shown strong potential in dynamic decision-making and local obstacle avoidance in USV path planning, its practical application is still restricted by the dynamic characteristics of the platform, the uncertainty of the marine environment, and strict navigation rules and safety requirements. COLREGs establish a framework of basic rules for collision avoidance at sea. The core requirements of COLREGs include maintaining a regular lookout, timely assessing collision risk, and taking early and sufficient collision avoidance actions in typical encounters scenarios such as encounter, crossing, and pursuit, as well as narrow channels and navigable separation conditions. Compared with UAVs or ground mobile robots, USVs not only have to deal with external disturbances such as wind, waves, and currents but also have to take into account trajectory smoothness, control feasibility, and real-time response capabilities.

USV path planning is essentially a multi-constraint, multi-objective, and strongly coupled decision-making process. Therefore, reinforcement learning in this scenario cannot directly apply the analysis framework of other unmanned platforms and must be adjusted in terms of state representation, action design, reward construction, and safety verification. Based on the repeated core difficulties in existing research, this section systematically summarizes the main constraints and challenges faced by reinforcement learning for USV path planning in practical applications.

### 3.1. The Paradox of the Reward Mechanism and the Problem of Mathematical Representation

In the local path planning of USVs, the reward function design is always one of the core problems faced by reinforcement learning methods. Since the task usually involves multiple objectives, such as collision avoidance safety, trajectory tracking, and energy consumption control at the same time, most of the existing studies integrate these heterogeneous indicators into a single scalar reward in a linear weighted manner to guide the training and convergence of the policy network. However, although this approach is concise in form, it is difficult to really deal with the priority conflicts between different objectives, especially in scenarios where there is obvious tension between security constraints and performance optimization; its limitations are more prominent.

This problem is particularly evident under the COLREGs constraint. The emphasis of COLREGs is not on performance compromises in the general sense but on navigation rules and safety boundaries with clear boundaries. If the safety of collision avoidance is simply transformed into the penalty term in the reward function, the safety boundary will degenerate from “a constraint that must be observed” to “a variable that can be weighed”. In this mechanism, the agent may test at the risk edge or even form a decision pattern close to the physical limit in order to pursue a higher cumulative reward. This shows that the linear weighted reward mechanism still has obvious shortcomings in describing the rigid requirements of maritime navigation safety, especially in the high-risk collision avoidance task, and its effectiveness and rigor are still worthy of further review [17,40].

This limitation comes not only from the way the reward term is constructed, but also from the fact that it often relies more on mathematical representations than on understanding the semantics of the task. Algorithms can optimize numerical goals, but they do not naturally distinguish between “optimizable performance” and “unbreakable safety boundaries.” At the same time, due to the significant dynamics and scene dependence of the marine environment, the reward mechanism with fixed weight is often difficult to adapt to the environment switching from open water to narrow channels and from smooth flow fields to complex flow fields. Existing methods still highly rely on expert experience to repeatedly debug the weight ratio, and lack mechanisms that can adaptively adjust the feedback logic according to the environmental context [8,23]. Therefore, the key question is not just “how to set the weights”, but whether the reward mechanism itself has the ability to express safety intentions and task preferences across scenarios.

### 3.2. Learning Stagnation and Lack of Robustness in Large-Scale Environments

As USV planning tasks expand from local areas to large-scale and long-range sea areas, the limitations of reinforcement learning in sample efficiency and reward sparsity gradually become apparent. Compared to the local scene, the effective feedback in the marine environment is sparser, and the agent needs to gradually identify a small number of directional signals in a large number of invalid explorations. Therefore, in the early stage of training, the search efficiency is often low, the strategy update is slow, and it is even difficult to form effective convergence [41]. Although previous studies have tried to introduce methods such as hierarchical decision-making, curriculum learning, or prioritized experience replay to improve learning efficiency, the computational overhead increases with the expansion of state space and the increase of task complexity, and the practical effect is still limited. The difficulty brought by a large-scale environment is not only reflected in the long training time but also reflected in the fact that the learning process itself is difficult to steadily advance.

In addition to training efficiency, the implementation stability of the strategy in real sea conditions also faces challenges. The state information obtained by USVs during actual navigation is often affected by sensor errors, communication delays, and environmental occlusion. For neural network strategies that rely on high-dimensional inputs, such disturbances easily affect their discrimination results on the environment, leading to fluctuations in control behaviors and even unnecessary action switching [33]. Especially in dynamic scenarios such as multi-ship encounters, narrow waterways, and complex flow fields, if the environmental representation fails to accurately highlight the key risk factors, the strategy model is more prone to misjudgment, making it difficult to maintain continuous and predictable navigation behavior.

Therefore, the key issue of reinforcement learning in maritime scenarios is not only whether it can learn an effective policy but also whether the policy can maintain stable execution in the complex and uncertain real environment. Due to the obvious time-varying and scene-dependent marine environment, it is often difficult to balance the learning efficiency and execution robustness by simply relying on a fixed structure and static feedback mechanisms. How to improve the model’s ability to adapt to perceptual disturbances and environmental changes without significantly increasing the training costs is still an important issue that restricts its engineering application.

### 3.3. Heterogeneity of Physical Constraints and Trust Crisis of Real Machine Migration

There are also clear differences between the simulated environment and the real ship dynamics. In order to take into account the computational efficiency, the existing research usually adopts a simplified three-degree-of-freedom motion model, which ignores the inertial lag and hydrodynamic coupling characteristics of USVs as an underactuated system when executing rudder angle commands [30]. This simplification has a relatively limited impact in low speed and smooth sea conditions, but when the sailing speed is increased or the sea state is complex, the model error is often further amplified, and the collision avoidance strategy learned in simulation shows path overshooting, control hysteresis, or action instability when deployed to the real ship. Although dynamic modeling reduces the training complexity, it also weakens the true transferability of the policy.

The reliability problem of the end-to-end reinforcement learning method itself has gradually become prominent. Such methods usually rely on neural networks to directly map states and actions. Although they have strong expressive power, their decision-making process lacks a clear physical explanation and is difficult to provide a strict stability guarantee [34]. For maritime scenarios, this issue is particularly sensitive because ship navigation requires not only the completion of path planning but also that basic requirements such as collision avoidance, safety, and controllable operation must be met. Once the system has abnormal behavior in extreme scenarios, it is often difficult to explain the reasons for the policy failure and determine whether the policy meets the security boundaries required for actual deployment by postmortem analysis alone. Therefore, the black-box features not only affect the interpretability of the model but also directly relate to the engineering acceptability of the method.

From the perspective of practical application, it is far from enough to rely only on the performance improvements in simulation if USVs’ intelligent navigation truly enters the complex sea areas. The marine environment itself has the characteristics of strong uncertainty, rapid scene changes, and many interference factors. If the model lacks explicit modeling of physical constraints, it is difficult to maintain stable performance under different working conditions. A more reasonable direction is to introduce mechanisms consistent with ship dynamics and safety constraints into the learning framework so that the policy has better interpretability and verifiability while maintaining a certain degree of flexibility. This balance is often more critical for USV systems oriented toward real deployment than simply pursuing training performance.

The core bottleneck faced by the above USVs’ reinforcement learning path planning is shown in Table 3.

## 4. Future Outlook

### 4.1. Offline Reinforcement Learning and Big Data-Driven Policy Evolution

Aiming at the problems of high risk and low learning efficiency of online learning in practical maritime deployment, offline reinforcement learning is now considered a feasible path to bridge the distance between theoretical research and practical application. Researchers have found that with the help of a large amount of navigation data accumulated over the history of manned ships or safe trajectories extracted from high-fidelity simulation environments, agents can first learn some robust decision logic without direct contact with the real environment. In this way, the risk of collision caused by random probing during early exploration can be avoided [45]. In addition, by introducing some conservative value estimation methods into the model or designing a more reasonable reward distribution mechanism, the problem of overly optimistic behavior caused by “distribution shift” can be effectively suppressed so as to improve the safety of unmanned vessels at the beginning of deployment in unfamiliar waters.

In the future, research may increasingly turn to a hybrid model of “offline pre-training-online fine-tuning”. In this method, the model is trained with basic skills such as obstacle avoidance in the offline stage, and then it is gradually adapted to the hydrological characteristics or dynamic environment of a specific sea area through safe online interaction [26]. As mentioned in positive studies, this gradually optimized learning architecture can not only accelerate the convergence speed of the model but also enable the USVs to have stronger adaptability in the face of seasonal sea state changes or cargo loading adjustments [9]. As the global shipping big data platform becomes more and more perfect, reinforcement learning based on real navigation trajectories in the future is also expected to break away from the dependence on simulation environments and move toward a development path that is closer to reality and more data-driven.

### 4.2. Deep Fusion of Neural Dynamics Driven by Physical Information

In order to alleviate the prediction bias between the simulation environment and the real world from the source, the introduction of physically informed neural networks (PINNs) is promoting the transformation of local planning algorithms from purely data-driven methods to taking into account physical consistency [45]. Some researchers have pointed out that if the ship kinematics equations, such as the classical Fossen 6-DOF model, are embedded into the policy network as mandatory constraints, each control command output by the algorithm can satisfy the basic hydrodynamic laws, thus avoiding the occurrence of “illegal solutions” that are not true in practice [34]. This deep fusion method can not only correct the behavior deviation of the neural network under extreme working conditions but also enable the model to still output the rudder angle command consistent with physical intuition when encountering large waves or strong winds [17].

In the future, the combination of physical laws with deep learning models may lead to more predictive neurodynamic architectures. For example, by embedding a differentiable physical simulator into the process of policy evaluation, the agent can evaluate how much load the current motion state will bring to the main engine or servo in advance while planning the path, and then actively avoid those dangerous operations that may lead to servo saturation or even capsize risk [35]. This reinforcement learning framework with physical boundary perception is expected to significantly improve the survival ability of USVs in narrow channels or harsh sea conditions so that the system can truly achieve high-level autonomous decision-making of “understanding physics and knowing limitations”, thereby pushing the actual combat ability of intelligent control further.

### 4.3. Interpretable Architecture and Layered Security Shell Mechanisms

In the face of industry doubts about the transparency and compliance of “black box” algorithms, the development of reinforcement learning architectures with interpretability is gradually becoming an important direction to improve system trustworthiness. The hierarchical critic evaluation system is introduced to decompose the complex collision avoidance decision into several sub-tasks that can be observed and traced. In this way, the shipping regulator can more clearly understand the motivation behind the algorithm issuing a certain instruction in a specific ship rendezvous scenario. Coupled with the visual attention mechanism and weight analysis, the operator can intuitively see how the algorithm determines the risk of a dynamic obstacle. This transparency in cognition makes human-machine collaboration more trustworthy and also provides some psychological and technical support for USVs to enter busy waters [38].

In terms of low-level security, the mainstream idea in the future may be to add a harder control loop to the reinforcement learning system. For example, the safety shell based on formal verification or a Control Barrier Function (CBF) was introduced so that each instruction generated by the algorithm had to pass a strict physical safety check before it was actually sent to the steering engine or the host computer. If there is a risk of collision, this safety mechanism can step in and take over control, bringing the system back to a safe state. In addition, some people think of using Large Language Models (LLMs) to parse those vaguely written maritime rules and inject more commonsense legal constraints into reinforcement learning so that autonomous planning systems can not only calculate the optimal solution but also take into account the ethical and legal boundaries in the real world.

## 5. Conclusions

Reinforcement learning technology in the field of unmanned surface vehicle path planning is undergoing a transition stage from theoretical verification to industrial application. Deep reinforcement learning has shown the potential to surpass traditional methods due to its ability to deal with complex nonlinear dynamic obstacles and multiple constraint optimization. However, there are still challenges such as imperfect reward function design, low sample efficiency, and the difference between simulation and real ships, which limit the reliability of its large-scale deployment and practical application. To achieve real engineering implementation, it is necessary to break through the pure data-driven black box thinking and actively integrate navigation rules and physical knowledge to improve the safety and interpretability of decision-making.

In the future, autonomous planning of unmanned surface vehicles will gradually shift from pure perception driven to a new paradigm of integration of perception and rationality. Relying on the deep refinement of massive navigation data through offline reinforcement learning, combining a physical information neural network to strengthen physical constraints, and ensuring the robustness of decision boundaries through hierarchical security policies, the system will improve adaptability in the actual environment while ensuring compliance and security [42]. With the continuous progress of algorithm transparency and physical simulation levels, reinforcement learning is expected to play a more central role in the field of intelligent shipping, leading the shipping industry to a digital, green, and intelligent future.

## Figures and Tables

**Table 1:** Comprehensive comparison of reinforcement learning algorithms for the local path planning of USVs.

Algorithm	Advantages	Disadvantages	Typical Scenario	References
DQN	The structure is mature, the utilization of offline samples is high, and the stability of static scenes is good.	The rudder angle output is discontinuous, the Q value is overestimated, and the “zigzag” trajectory is easy to produce.	Scenes with discrete action space and regular environmental structure, such as ports and inland rivers.	[12,26,39]
PPO	The training is smooth, the path smoothness is high, and the robustness to multiple complex constraints is strong.	The sampling efficiency is low, there is a dependence on online data, and the convergence period is relatively long.	Scenarios requiring continuous control, dynamic collision avoidance, and satisfying COLREGs constraints, such as narrow channel passage and rule-constrained navigation.	[3,14,29]
SAC	Strong generalization, robust to environmental noise (wind, wave, and current) interference ability outstanding.	The computational overhead is large and may produce excessive yaw exploration in deterministic tasks.	Scenarios with strong uncertainty and complex disturbances, such as unknown waters and strong wind, wave, and current disturbance environments.	[9,27,30]
TD3	The estimation bias is significantly suppressed, and the high-precision control performance of the underactuated system is excellent.	It is sensitive to the accuracy of state feedback, and the convergence efficiency decreases in the perceptual fuzzy environment	Trajectory tracking, high-precision continuous control, and Sim-to-Real migration scenarios for underactuated USVs.	[33,34,35]
Hybrid architecture	It takes into account global optimality and local flexibility to solve the sparse reward problem of reinforcement learning.	The architecture design is complex, and it is easy to produce decision redundancy or conflict between each layer’s logic.	Complex waterway scenarios that require both global feasibility and local flexible responses are required.	[36,37,38]

**Table 2:** Five-dimensional performance comparison of representative reinforcement learning and hybrid planning methods for USV local path planning.

Method Category	Path Smoothness	Dynamic Adaptability	Convergence Stability	Sample Efficiency	Real-Time Performance	References
DQN Family	Moderate	Moderate	Moderate to High	High	Moderate	[5,12,18,25,26,29,39,40]
PPO Family	High	High	High	Moderate	Moderate	[3,14,16,17,25,28,29]
SAC Family	High	Very High	High	Moderate	Moderate	[9,12,20,27,28,30]
TD3 Family	High	High	High	Moderate	Moderate to High	[15,33,34]
Hybrid Planning (RL+A*/RRT*/MPC/APF/DWA)	Very High	Very High	High	Moderate to High	Moderate to High	[28,30,31,35,39,41,42]

**Table 3:** Key challenges and bottlenecks in local path planning of reinforcement learning for USVs.

Constraints	The Core Problem	Negative Effects	References
Reward mechanism layer	Reward sparsity and the mechanism paradox	In large-scale sea areas, the agent may receive effective feedback only after a long delay, resulting in extremely slow training convergence. Excessively harsh punishments often lead to “overconservatism” or “risk-taking” due to the imbalance between rewards and punishments.	[33,34,43,44]
Sample efficiency layer	Sample inefficiency in a large-scale environment	In dynamic multi-obstacle environments, the policy search space grows exponentially. It is difficult to cover all edge cases by relying solely on on-policy training, resulting in a long training cycle.	[38]
Real machine deployment layer	Sim-to-Real migration crisis	The simulation ignores the time-varying disturbances of wind, waves, and current in the real sea state and the nonlinear hysteresis of the propeller, which leads to a decline in the accuracy of obstacle avoidance, the severe oscillation of the track, and the trust crisis in the application of the model in real aircraft.	[30]
Black box decision layer	Interpretability and security of algorithms	It is difficult to hardcode industry logic such as Maritime Conventions (COLREGs) into neural networks. In the emergency conflict scenarios, the obstacle avoidance instructions output by the system lack physical logic support, which is difficult to meet the requirements of shipping safety regulations.	[17,45]

## Data Availability

No new experimental data were created or analyzed in this study. All data used in this review are publicly available in the cited references. For further details, please refer to the original publications cited throughout the manuscript.

## Abbreviations

The following abbreviations are used in this manuscript:

USVs	Unmanned Surface Vehicles
APF	Artificial Potential Field
RRT	Rapidly-exploring Random Tree
COLREGs	International Regulations for Preventing Collisions at Sea
RL	Reinforcement Learning
DRL	Deep Reinforcement Learning
MODQN	Multi-target Deep Q-Network
DQN	Deep Q-Network
PPO	Proximal Policy Optimization
SAC	Soft Actor-Critic
RBF	Radial Basis Function
CRI	Collision Risk Index
HMC	Hamiltonian Monte Carlo
TD3	Twin Delayed Deep Deterministic Policy Gradient
DDPG	Deep Deterministic Policy Gradient
PINNs	Physically Informed Neural Networks
CBF	Control Barrier Function
LLMS	Large Language Models
